# Detection of novel and potentially actionable anaplastic lymphoma kinase (*ALK*) rearrangement in colorectal adenocarcinoma by immunohistochemistry screening

**DOI:** 10.18632/oncotarget.4462

**Published:** 2015-07-01

**Authors:** Jeeyun Lee, Hee Cheol Kim, Jung Yong Hong, Kai Wang, Sun Young Kim, Jiryeon Jang, Seung Tae Kim, Joon Oh Park, Ho Yeong Lim, Won Ki Kang, Young Suk Park, Jiyun Lee, Woo Yong Lee, Yoon Ah Park, Jung Wook Huh, Seong Hyeon Yun, In-Gu Do, Seok Hyung Kim, Sohail Balasubramanian, Philip J. Stephens, Jeffrey S. Ross, Gang Gary Li, Zachary Hornby, Siraj M. Ali, Vincent A. Miller, Kyoung-Mee Kim, Sai-Hong Ignatius Ou

**Affiliations:** ^1^ Department of Medicine, Division of Hematology-Oncology, Samsung Medical Center, Sungkyunkwan University School of Medicine, Seoul, Republic of Korea; ^2^ Department of Surgery, Samsung Medical Center, Sungkyunkwan University School of Medicine, Seoul, Republic of Korea; ^3^ Department of Internal Medicine, Chung-Ang University College of Medicine, Dongjak-Gu, Seoul, Republic of Korea; ^4^ Foundation Medicine Inc, Cambridge, Massachusetts, USA; ^5^ Department of Pathology and Translational Genomics, Samsung Medical Center, Sungkyunkwan University School of Medicine, Seoul, Republic of Korea; ^6^ Department of Pathology and Laboratory Medicine, Albany Medical College, Albany, New York, USA; ^7^ Ignyta Inc, San Diego, California, USA; ^8^ Innovative Cancer Medicine Institute, Samsung Medical Center, Seoul, Korea; ^9^ Chao Family Comprehensive Cancer Center, University of California Irvine School of Medicine, Orange, California, USA

**Keywords:** colorectal carcinoma, anaplastic lymphoma kinase (ALK) rearrangement, immunohistochemistry, next generation sequencing

## Abstract

**Purpose:**

Anaplastic lymphoma kinase *(ALK)* rearrangement has been detected in colorectal carcinoma (CRC) using advanced molecular diagnostics tests including exon scanning, fluorescence *in situ* hybridization (FISH), and next generation sequencing (NGS). We investigated if immunohistochemistry (IHC) can be used to detect *ALK* rearrangement in gastrointestinal malignancies.

**Experimental designs:**

Tissue microarrays (TMAs) from consecutive gastric carcinoma (GC) and CRC patients who underwent surgical resection at Samsung Medical Center, Seoul, Korea were screened by IHC using ALK monoclonal antibody 5A4. IHC positive cases were confirmed by FISH, nCounter assays, and NGS-based comprehensive genomic profiling (CGP). ALK IHC was further applied to CRC patients enrolled in a pathway-directed therapeutic trial.

**Results:**

Four hundred thirty-two GC and 172 CRC cases were screened by IHC. No GC sample was ALK IHC positive. One CRC (0.6%) was ALK IHC positive (3+) that was confirmed by *ALK* FISH and a novel *CAD-ALK (C35; A20)* fusion variant that resulted from a paracentric inversion event inv(2)(p22–21p23) was identified by CGP. One out of 50 CRC patients enrolled in a pathway-directed therapeutic trial was ALK IHC positive (3+) confirmed by *ALK* FISH and found to harbor the *EML4-ALK (E21, A20)* fusion variant by CGP. Growth of a tumor cell line derived from this *EML4-ALK* CRC patient was inhibited by ALK inhibitors crizotinib and entrectinib.

**Conclusions:**

ALK IHC is a viable screening strategy for identifying *ALK* rearrangement in CRC. *ALK* rearrangement is a potential actionable driver mutation in CRC based on survival inhibition of patient tumor-derived cell line by potent ALK inhibitors.

## INTRODUCTION

The multi-targeted anaplastic lymphoma kinase (ALK) inhibitor crizotinib has demonstrated significant improvement in progression-free survival (PFS) over chemotherapy as both first-line or second-line treatment of *ALK*-rearranged non-small cell lung cancer (NSCLC) [1, 2] and has firmly established that *ALK* rearrangement is a targetable driver mutation in NSCLC. *ALK* breakapart fluorescence *in situ* (FISH) was until recently the only companion diagnostic assay approved by the US Food and Drug Administration (FDA) for the detection of *ALK* rearrangement [3]. ALK IHC has been approved as a companion diagnostic kit in other countries such as China and Taiwan and in the US in June, 2015.

*ALK* rearrangement has also been identified in 0.4% to 2.5% of colorectal carcinoma (CRC) by exon array profiling [4], fluorescence *in situ* hybridization (FISH) [5], and next generation sequencing (NGS) [6] assays performed on archival tumor specimens. Given the relative low incidence of *ALK* rearrangement in CRC and the unknown clinical significance of this rearrangement in CRC, a routine and cost-effective diagnostic assay is needed to allow broad screening for *ALK* rearrangement in CRC and identify these patients for potential enrollment into clinical trials. ALK immunohistochemistry (IHC) has been shown to be sensitive and specific and cost effective to screen for *ALK* rearrangement in NSCLC [7]. Given that both *ALK* and *ROS1* rearrangements have been identified in CRC [5] and we have previously identified *ROS1* rearrangement in GC [8], we performed a screening study for *ALK* rearrangement in GC and CRC using ALK IHC.

## RESULTS

### Patient characteristics

A total of 172 CRC and 432 GC patient samples were analyzed by ALK IHC. Primary site of CRC was colon in 100 patients (58.1%) and rectum in 72 patients (41.9%) (Table 1). For the GC patients group, slightly more than half of patients (53.3%) presented with distal GC (Table 2).

**Table 1:** Characteristics of the colorectal adenocarcinoma patients screened (*N* = 172)

Characteristic	Number	(%)
**Age (years)** Median, (range)	59	(28–84)
**Sex** Male Female	103 69	(59.9) (40.1)
**Primary site** Colon Rectum	100 72	(58.1) (41.9)
**Operation** Hemicolectomy Low anterior resection Anterior resection Mile's operation Others	54 45 44 23 6	(31.4) (26.2) (25.6) (13.4) (3.5)
**Pathologic stage** I II III IV	20 73 76 3	(11.6) (42.4) (44.2) (1.7)
**Gross type** Ulceroinfiltrative Fungating	151 21	(87.8) (12.2)
**Differentiation** Well to moderately differentiated Poorly differentiated	161 11	(93.6) (6.4)
**Lymphovascular invasion** Positive Negative	25 147	(14.5) (85.5)
**Neural invasion** Positive Negative	4 168	(2.3) (97.7)
**Adjuvant treatment** Chemotherapy alone Radiotherapy alone Both chemotherapy and radiotherapy None	79 0 44 49	(45.9) (0.0) (25.6) (28.5)

**Table 2:** Characteristics of the gastric carcinoma patients screened (*N* = 432)

Characteristics	*N* = 432
**Age (yrs)** Median, range	53, 23–74
**Sex** Male Female	280 (64.8%) 152 (35.2%)
**Type of gastrectomy** Subtotal gastrectomy Total gastrectomy Others	256 (59.3%) 175 (40.5%) 1 (0.2%)
**Location of tumor** Distal 1/3 Middle 1/3 Cardia, GE junction Whole, multicentric Reminant stomach	231 (53.5%) 130 (30.1%) 53 (12.3%) 17 (3.9%) 1 (0.2%)
**Grade** Well/moderate differentiated tubular Poorly-differentiated tubular Signet ring cell Mucinous Papillary Hepatoid Others	111 (25.7%) 200 (46.3%) 101 (23.4%) 14 (3.2%) 3 (0.7%) 2 (0.5%) 1 (0.2%)
**Lauren type** Intestinal Diffuse Mixed	139 (32.2%) 280 (64.8%) 13 (3.0%)
**Lymphovascular invasion** Present Not present	212 (49.1%) 220 (50.9%)
**pT1 stage** T1 T2 T3	38 (8.8%) 317 (73.4%) 77 (17.8%)
**pN stage** N0 N1 N2 N3	40 (9.3%) 223 (51.6%) 103 (23.8%) 66 (15.3%)
**AJCC stage** IB II IIIA IIIB IV	68 (15.7%) 167 (38.7%) 111 (25.7%) 19 (4.4%) 67 (15.5%)

### Identification of a novel CAD-ALK fusion variant

One rectal adenocarcinoma patient (0.6%) showed intense, granular staining (3+) by ALK IHC. ALK protein expression was observed in the cytoplasm of tumor cells with diffuse staining pattern in both differentiated and undifferentiated carcinoma areas with strong intensity (Figure 1A). *ALK* FISH revealed 25% of tumor cells had red and green signals that were two or more signal diameters apart were observed (Figure 1B). The nCounter assays demonstrated the loss of 5′portion of the *ALK* gene (Figure 1C) but failed to detect fusion partner gene using the selected fusion gene sets of *KIF5B*, *EML4*, *KLC1*, *SMCF1* and *C2orf44*.

**Figure 1A:**
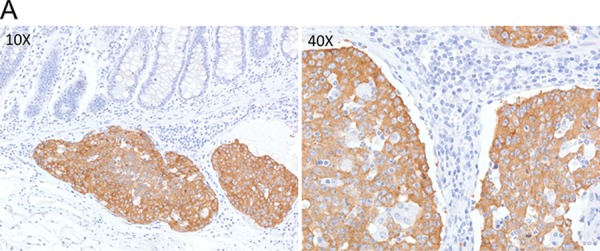
ALK IHC (3+) staining in a rectal adenocarcinoma patient tumor sample Low power field examination (10X; Left) shows strong cytoplasmic staining in cancer cells compared to control normal colonic crypts. High power examination (40X) shows dense brown granular cytoplasmic staining.

**Figure 1B:**
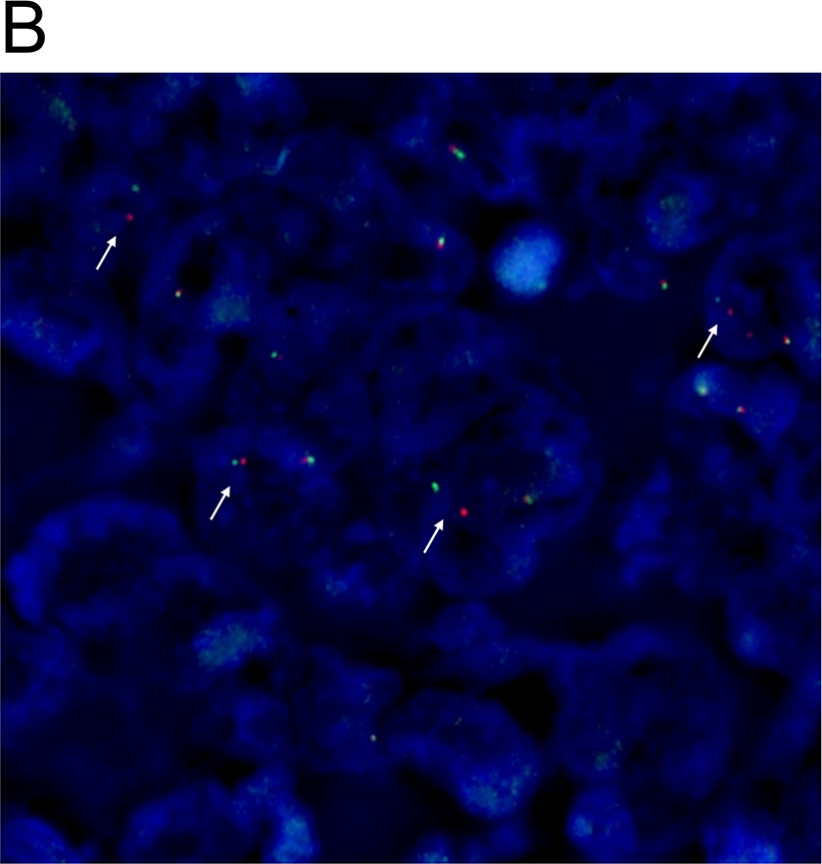
Detection of *ALK* rearrangement by *ALK* break-apart by fluorescence *in situ* hybridization (FISH) in the ALK IHC (3+) rectal adenocarcinoma patient (white arrows)

**Figure 1C:**
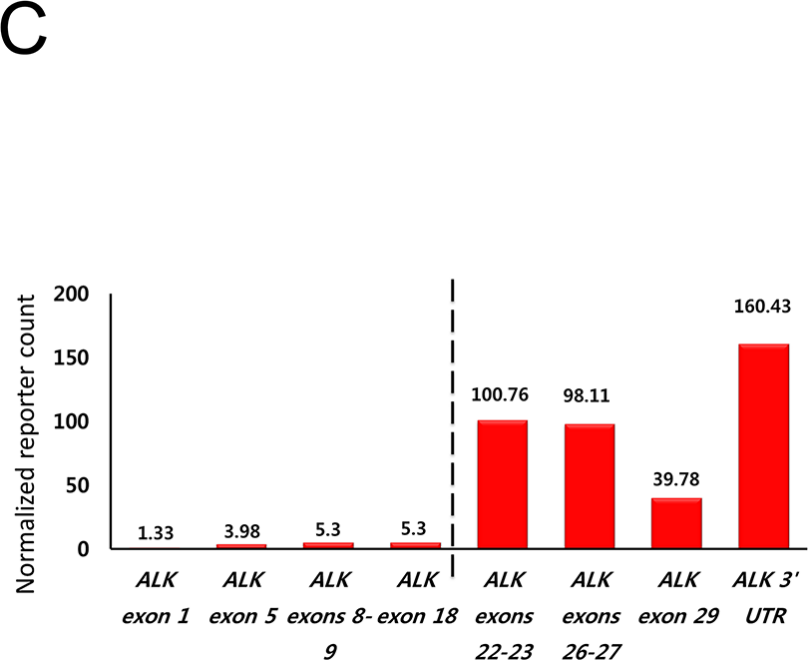
Nanostring 3′/5′ ratio of *ALK* reporter readout indicating the loss of the 5′portion of *ALK* gene

A novel *CAD-ALK* fusion variant was identified by CGP in this patient case. The *CAD* (Carbamoyl-phosphate synthetase 2, Aspartate transcarbamylase, and Dihydroorotase) gene is located on chromosome 2p21–22 and contains 45 exons [15] and is transcribed in the opposite direction as *ALK* (Figure 2A). The *CAD-ALK* fusion variant is generated by an intra-chromosomal inversion event fusing the exons 1–35 of *CAD* to exons 20–29 of *ALK* (Figure 2A). The full-length CAD protein is comprised of 2, 225 amino acids and is a “multifunctional” protein responsible for four enzymatic activities of the pyrimidine pathway (gluymine amidotransferase [GATase], carbamoly-phosphate synthase [CPSase], dihydroorotase [DHOase], and aspartate transcarbamylase [ATCase]) (Figure 2B). The CAD-ALK fusion variant results in the first 1864 amino acids of CAD, which includes the GATase, CPSase, and DHOase enzymes but not the ATCase domains, fused to the full length kinase domain of ALK (Figure 2B). Both *KRAS* and *BRAF* were wildtype by CGP (Table 3) and no additional kinase fusions were identified.

**Figure 2A:**
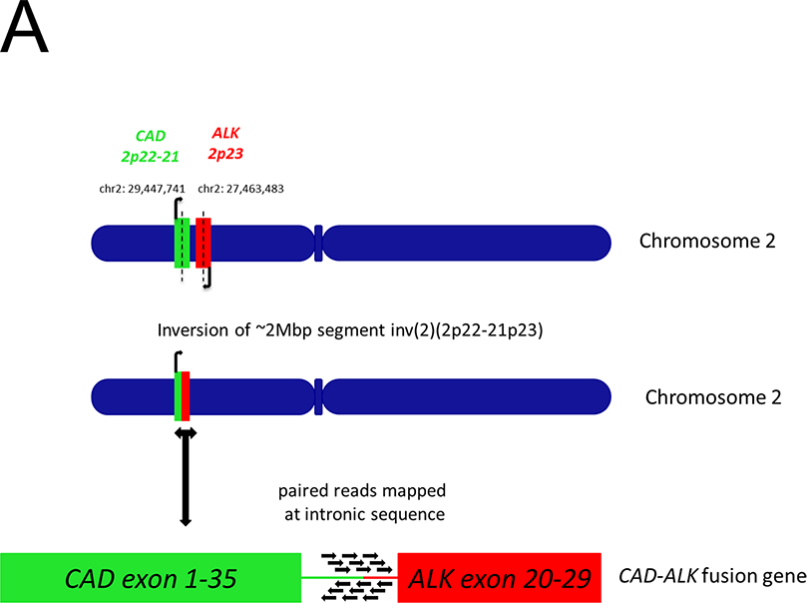
Schematic of chromosomal location and transcription direction and breakpoint of *CAD* and *ALK* genes in the *CAD-ALK* positive CRC patient

**Figure 2B:**
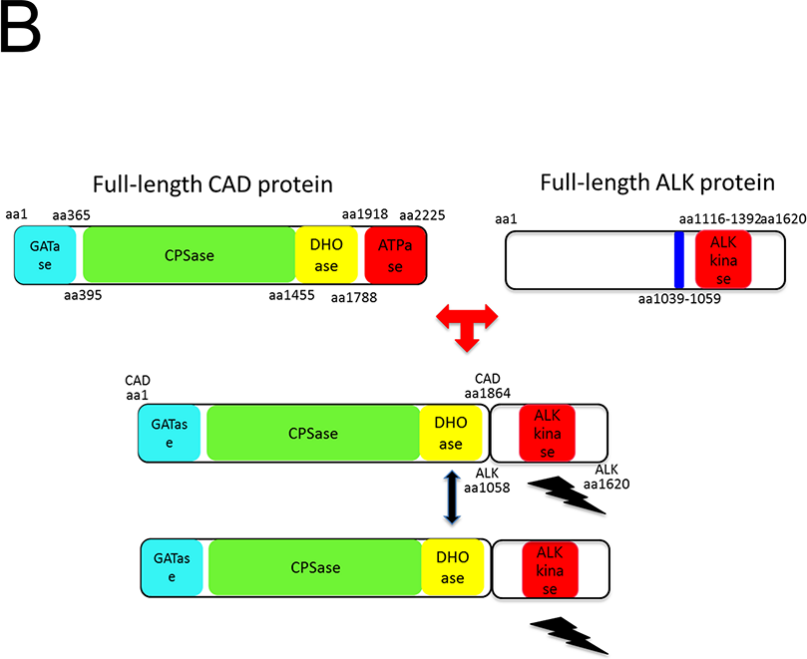
Schematic of the CAD-ALK fusion protein domains and potential dimerization domains

**Table 3:** Comparison of the methods and clinicopathologic characteristics of *ALK*+ CRC patients identified from this study and in the literature

Study	Aisner et al. [5]	Huoang et al. [17]	This report	This report
Frequency	1/236* (0.4%)	1/1889 (0.05%)	1/172 (0.6%-total) 1/72 (1.4%-rectal)	1 out of 50 CRC (2%)
Screening Method	FISH	IHC (5A4 antibody)	IHC (5A4 antibody)	IHC (5A4 antibody)
Confirmation method	RT-PCR	FISH (Vysis Abbott Molecular)	FISH (Vysis Abbott Molecular), NGS (Foundation Medicine)	FISH (Vysis Abbott Molecular), Nanostring, NGS (Foundation Medicine)
Source of tissue	TMA	TMA	TMA	Patient tumor tissue
Location	Rectum	Colon (distal transverse)	Rectum	Colon
Age	84	87	46	65
Ethnicity	Australian	Australian	Korean	Korean
Gender	Female	Female	Female	Male
ALK Fusion partner	EML4	NR	CAD	EML4
ALK fusion variant	*EML4-ALK* (E6, A20)	NR	*CAD-ALK* (C35; A20)	*EML4-ALK* (E21; A20)
Histologic differentiation	NR	NR	Poor	Poor
Signet ring features	No	No	No	No
Site of metastasis	Lymph nodes, lung	None	Lymph nodes, lung,pericardium	Mediastinal/cervical lymph nodes
Pattern of expression of ALK rearrangement	Scattered, in areas of high grade dysplasia, with intratumoral heterogeneity	Diffuse	Diffuse, cytoplasmic	Diffuse
*KRAS* status	KRAS G12A	Wildtype	Wildtype	Wildtype
*BRAF* status	NR	Wildtype	Wildtype	Wildtype

* 236 out of 286 tumor samples evaluable;

CAD: carbamoyl-phosphate synthetase 2, aspartate transcarbamylase, and dihydroorotase; EML4: echinoderm microtubule associated protein like 4;

IHC: immunohistochemistry; FISH: fluorescence *in situ* hybridization; MSI: microsatellite instability; MSS: microsatellite stable NA; not applicable; NGS; next generation sequencing; NR: not reported; RT-PCR: reverse transcriptase-polymerase chain reaction; TMA: tissue microarray;

The CAD-ALK patient was a 46 years old Korean woman who underwent low anterior resection and the pathology revealed a 6.5 cm poorly-differentiated adenocarcinoma with perirectal soft tissue extension and metastasis in 4 out of 34 regional lymph nodes (AJCC stage IIIB). She received adjuvant 5-fluorouracil (5-FU)-based concurrent chemoradiation but developed metastatic disease shortly and died of pericardial tamponade 2 months later. In the remaining CRC and all of the GC cases, ALK protein expression was not observed.

### Identification of an *EML4-ALK* rearranged colon cancer patient

One out of a total of 50 CRC patients enrolled in the NEXT-1 trial was screened positive by ALK IHC. This patient is 65-year old male patient who initially presented with stage IV colon adenocarcinoma with multiple retroperitoneal lymph nodes and supraclavicular lymph nodes (Figure 3A). After 12 cycles of cetuximab/FOLFIRI (5-fluorouracil, leucovorin, irinotecan) chemotherapy, the patient underwent palliative surgical resection of colon cancer which revealed poorly differentiated adenocarcinoma, demonstrated diffuse and intense ALK staining (3+) by ALK IHC (Figure 3B). *ALK* FISH was positive with 50% cells demonstrating breakapart signals (Figure 3C). CGP revealed *EML4-ALK (E21; A20)* fusion variant. The tumor was found to be *KRAS* and *BRAF* wildtype (Table 3).

**Figure 3A:**
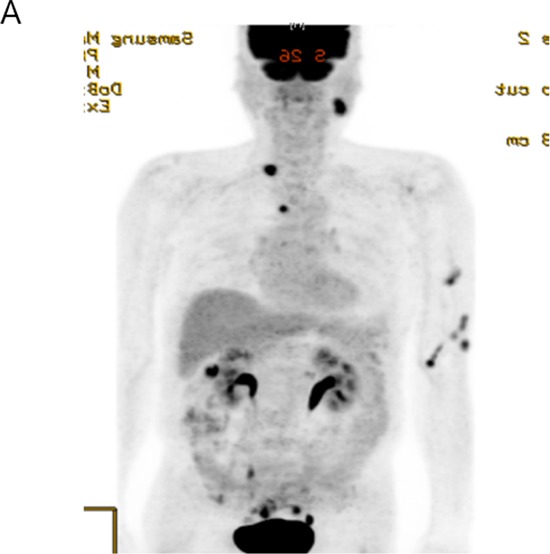
2-deoxy-2-(18F)fluoro-D-glucose positron emission tomography (PET) scan of the *EML4-ALK* CRC patient at the time of presentation demonstrating metastatic retroperitoneal and cervical lymph nodes metastasis

**Figure 3B:**
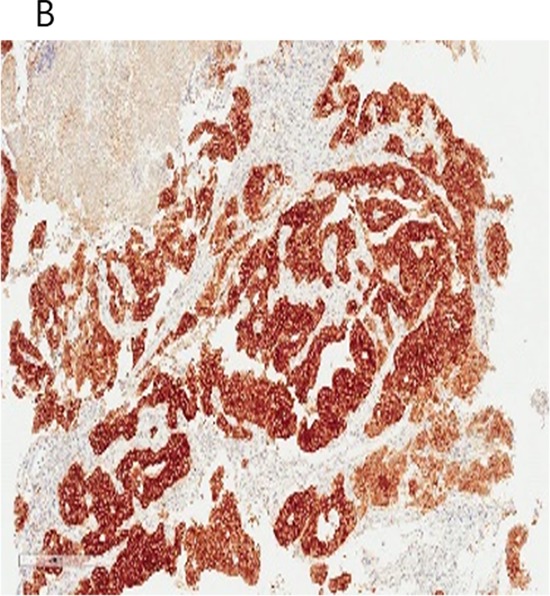
ALK IHC showing strong diffuse staining of ALK antibody (3+) of the *EML4-ALK (E21; A20)* adenocarcinoma of the colon patient after palliative resection

**Figure 3C:**
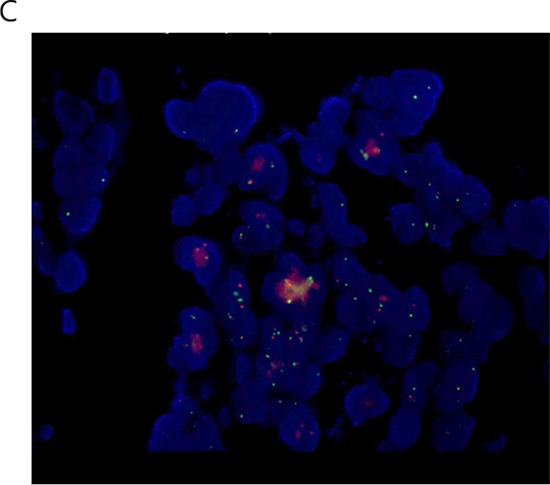
Break-apart *ALK* FISH of the *EML4-ALK (E21;A20)* colon patient demonstrating the presence of breakapart signals in 50% cells

Tumor cell line derived from this patient was inhibited by 1 μM crizotinib (*p* < 0.0001, one-way ANOVA test) or to a greater extent with 1 μM entrectinib (*p* < 0.0001 vs. no ALK inhibitor; *p* < 0.0001 vs. crizotinib one-way ANOVA test) (Figure 3D). Both crizotinib and entrectinib inhibited the phosphorylation of ALK and its downstream signal molecules, including ERK1/2 and AKT from this *EML4-ALK* rearranged colon patient tumor derived cell line (Figure 3E).

**Figure 3D:**
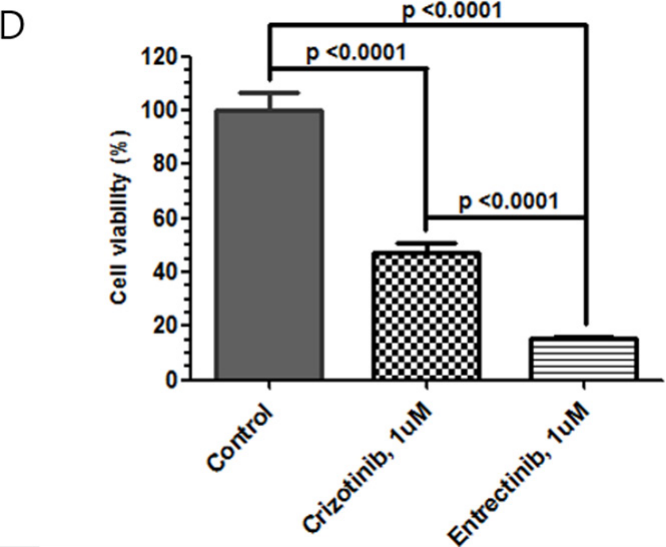
Inhibition of the growth of the *EML4-ALK* CRC patient derived tumor cells with 1 μM crizotinib or 1 μM entrectinib

**Figure 3E:**
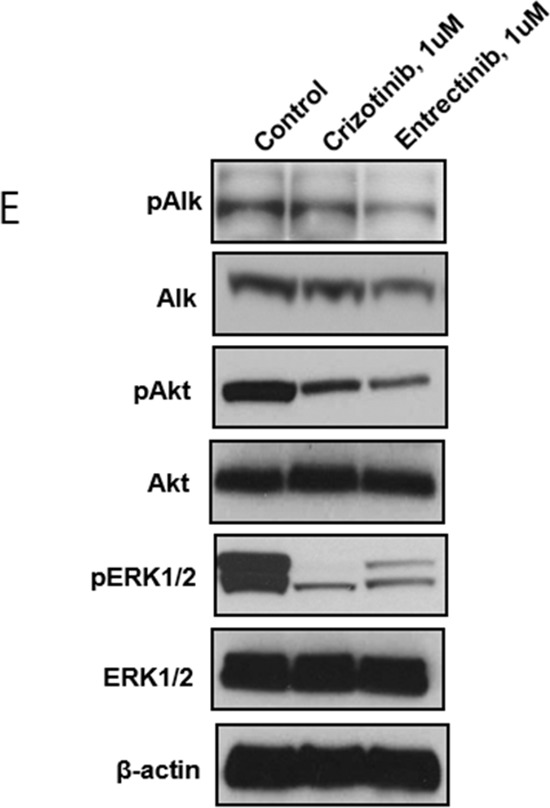
Western blot demonstrating Inhibition of the phosphorylation of the ALK protein and other downstream signal molecules in the *EML4-ALK* CRC patient tumor derived cell line after inhibition by crizotinib or entrectinib

## DISCUSSION

We demonstrated that IHC is a viable screening strategy for detecting *ALK* rearrangement in CRC and during the process identified a novel *CAD-ALK* fusion in CRC. The frequency of *ALK* rearrangement in CRC in this study was of 0.6% (1/172) among Korean CRC tumor samples. The discrepancy may be due to the TMAs were more than 10 years old which may affect the performance of the diagnostic assays. Lin and colleagues identified two *EML4*-*ALK* fusion CRC (2/83, 2.4%) and identified potentially up to 5 *ALK* rearranged CRC out of 83 (6%) CRC samples using an exon scanning method [4]. Aisner and colleagues identified one *EML4-ALK* CRC tumor out of 236 patient tumor samples (0.4%) using FISH as a screening assay (Table 3) [5]. Lipson and colleagues identified one *C2orf44-ALK (WDCP-ALK)* CRC patient among 40 CRC patients (2.5%) using CGP [6]. Chromosome 2 open reading frame 44 (*C2orf44*) was recently shown to encode WD repeat and coiled-coil containing protein (WDCP) [16]. Houang and colleagues performed a large scale screening for both *ALK* and *ROS1* rearrangements in CRC and found only one case among 1889 (0.05%) CRC cases was FISH positive (ALK IHC 3+), but no fusion partner was identified [17] (Table 3). The study by Houang and colleagues and our study indicated ALK IHC can be a very specific assay for detection of *ALK* rearrangement in CRC. Although signet-ring histology has been associated with *ALK*-rearranged NSCLC [18], none of the reports identified signet-ring histology in the colon adenocarcinoma [5, 6, 17]. Indeed Miller and colleagues have utilized signet ring histology as a screening criterion but failed to detect *ALK* rearrangement by break-apart FISH in upper gastrointestinal malignancies [19]. Given the low frequency of *ALK* rearrangement in CRC, a precise clinicopathologic profile of *ALK*-rearranged CRC remains to be determined. We were not able to detect *ALK* rearrangement in GC as there was no ALK IHC positivity among the 432 GC samples screened. It remains possible that *ALK* rearrangement exists but is likely to be extremely infrequent in GC.

We identified two different ALK fusions (*CAD-ALK, EML4-ALK*) in CRC in this report. *CAD* is located on chromosome 2p22-p21 in close proximity to *ALK* (and *EML4*) [15] and the transcription of *CAD* is in the opposite direction of *ALK* (Figure 2A). Thus the *CAD-ALK* fusion is likely generated by an intra-chromosomal inversion event similar to the event that generated *EML4-ALK* in NSCLC [20, 21]. Furthermore it has been shown that the DHoase domain, which is retained in the *CAD*-*ALK* fusion, can homo-multimerize with other CAD proteins [22] thus functioning analogously as the classical “coiled-coil” domain found in EML4 protein and leading to the aberrant activation of the ALK kinase [20, 21]. The *ALK*-rearranged CRC patient identified as part of the NEXT-1 trial harbors the *EML4-ALK* (E21:A20) where exons 1–21 of *EML4* is fused to exons 20–29 of *ALK* which has been reported only once in CRC [5] but so far has not been reported in *ALK*-rearranged NSCLC. Recently, suppressor of MEK1 homolog 2 (SMEK2)-ALK fusion variant *SMEK2-ALK* (*S11; A2*) has been reported in rectal adenocarcinoma, which fused the first 11 exons of *SMEK2* to exons 2–29 of *ALK*, independently providing further evidence the existence of *ALK* rearrangement in CRC [24].

The success of crizotinib in *ALK*-rearranged NSCLC [1, 2] has opened the door for potential treatment of other “ALKoma” [23] with crizotinib and other ALK inhibitors. There are ongoing clinical trials of ALK inhibitors that are organ agnostic [i.e. TSR-011 (NCT02048488); entrectinib (NCT02097810)]. The inhibition of tumor derived cell line from our *EML4-ALK* CRC patient by two separate ALK inhibitors is encouraging and provides pre-clinical rationale for enrolling *ALK*-rearranged CRC patients onto clinical trials with ALK inhibitor. ALK IHC has been automated for comprehensive screening for *ALK* rearrangement in NSCLC with 100% sensitivity and 98% specificity (98%) [25] and could be employed as the screening strategy for *ALK* rearrangement in CRC.

## MATERIALS AND METHODS

### Patients and tissue microarrays

The GC patient in this study has been described [9]. For the CRC patients included in this study, all received surgical resection with curative intent at Samsung Medical Center, Seoul, Korea, between January 2000 and December 2001. Inclusion criteria were as follows: (1) pathologically confirmed adenocarcinoma of stomach, colon and rectum; (2) adequate amount and quality of paraffin-embedded tumor blocks; (3) available data on clinicopathologic characteristics; (4) no prior chemotherapy or chemoradiation for GC or CRC. For tissue microarray construction, all H&E stained slides were reviewed and the representative area was carefully selected and marked on all paraffin blocks. A 3 mm tissue core was taken from the representative region of each tumor specimen using Accumax (ISU Abxis, Seoul, Korea) as previously described [8]. The study protocol was approved by Institutional Review Board of Samsung Medical Center.

### ALK immunohistochemistry (IHC)

ALK IHC was carried out on 3-μm thick tissue using a Ventana automated immunostainer (Ventana Medical Systems, Tucson, AZ) according to the manufacturer's protocol. Briefly, the slides were deparaffinized using EZ Prep (Ventana Medical Systems) at 75°C for 4 minutes and heat pretreatment at 100°C for 20 minutes. The antibody for ALK (mouse monoclonal, clone 5A4, Novocastra, Newcastle, United Kingdom) was diluted to 1:30 and incubated at 42°C for 2 hours. Signals were detected with an iView detection kit (Ventana Medical Systems) based on a streptavidin-biotin method. Mayer's hematoxylin was used for counterstaining for 2 minutes at room temperature. ALK expression was semiquantitatively assessed based on the intensity of staining and the proportion of stained cells and scored by two independent pathologists (IG Do and KM Kim). An IHC score was assigned as 0 (no staining), 1+ (faint cytoplasmic staining in ≤10% of tumor cells), 2+ (moderate, smooth cytoplasmic staining), and 3+ (intense, granular cytoplasmic staining). IHC scores of 2+ or 3+ were regarded as ALK IHC positive.

### *ALK* florescence *in situ* hybridization (FISH)

*ALK* FISH analysis was performed in ALK IHC positive cases. A commercially available break-apart probe specific to the ALK locus (Vysis LSI ALK Dual Color, break-apart probe; Abbott Molecular, Des Plaines, IL, USA) was used. FISH analysis was considered positive for *ALK* rearrangement if >15% of tumor cells (≥50 tumor cells counted) showed isolated red signals and/or split red and green signals. Interpretation of FISH analysis was performed by two independent pathologists (IG Do and KM Kim).

### ALK fusion transcript assay by nCounter anchored multiplex polymerase chain reaction assays

nCounter anchored multiplex polymerase chain reaction assays were performed according to the manufacturer's protocol (NanoString, Seattle, WA). Briefly, 500 ng of total RNA was hybridized to nCounter probe sets for 16 hours at 65°C. Samples were processed using an automated nCounter Sample Prep Station (NanoString Technologies, Inc., Seattle, WA). Cartridges containing immobilized and aligned reporter complex were subsequently imaged on an nCounter Digital Analyzer (NanoString Technologies, Inc.). Reporter counts were collected using NanoString's nSolver analysis software version 1, normalized, and analyzed as previously described [10]. This technology has been shown to successfully identify novel fusion partners to *ALK*- and *RET*-rearranged NSCLC [11, 12]. To identify fusion partners of ALK in CRC and GC, primers for *KIF5B*, *EML4*, *KLC1*, *SMCF1* and *C2orf44* were designed and used for analyses.

### NGS-based comprehensive genomic profiling (CGP) assay

Ten 4 μm unstained, formalin-fixed paraffin-embedded (FFPE) tissue slides of ALK IHC positive cases were sent to a Clinical Laboratory Improvement Amendments (CLIA)-certified and College of American Pathologists (CAP)-accredited laboratory (Foundation Medicine Inc, Cambridge, MA) for CGP testing. Macro-dissection to enrich specimens of ≥20% tumor content was performed as warranted. DNA was extracted from unstained FFPE sections and quantified by a Picogreen fluorescence assay. 50–100 ng of DNA was used for library construction. Hybridization capture of all coding exons of 405 cancer-related genes and selected introns of 31 genes frequently rearranged in solid tumors was performed. Hybrid-capture libraries were then sequenced to >500x average unique coverage with >100x at >99% of exons using Illumina HiSeq instrument. Sequencing data were processed using a customized analysis pipeline designed to detect all classes of genomic alterations, including base substitutions, short insertions and deletions, copy number alterations, and genomic rearrangements [13].

### Screening of patients for *ALK* rearrangement for a pathway-directed therapy trial

Between November 2013 to August 2014, eligible patients with metastatic solid tumors were enrolled into an institutional review board approved, pathway-directed therapy NEXT-1 (Next generation pErsonalized tX with mulTi-omics and preclinical model) trial; http://www.clinicaltrials.gov, NCT02141152) at Samsung Medical Center. ALK IHC was performed when sufficient tumor tissue were available. As part of the NEXT trial, patient derived tumor cell lines were isolated from tumor tissue in patients with targetable mutations after obtaining informed consent form.

### Culture and treatment of patient tumor derived cell lines

After tumors were surgically removed and homogenized, the cells were cultured in RPMI media supplemented with 10% fetal bovine serum, 0.5 μg/ml of hydrocortisone (Sigma Aldrich), 5 μg/ml of insulin (PeproTech, Rocky Hill, NJ, USA), 5 ng of EGF and FGF (PeproTech). Crizotinib was purchase from Selleck Chemical (Houston, TX, USA). Entrectinib was provided by Ignyta, Inc (San Diego, CA, USA) under a material transfer agreement. After pathologic confirmation, cells were seeded on 1∼2 × 10^6^ cells/10 mm dishes or 5000 cells/well/96well plate, treated with 1 μM of crizotinib or 1 μM of entrectinib (RXDX-101) and incubated for 72 hours at 37°C in a humidified atmosphere of 5% carbon dioxide for analysis of Immunoblotting and cell proliferation inhibition assay in triplicates. Entrectinib is an ALK, ROS1, NTRK1, NTRK2, NTRK3 inhibitor that is currently undergoing a phase 1/2 clinical trial in the US (http://www.clinicaltrials.gov; NCT02097810) and a first in human study in Italy [14]. Inhibition of tumor derived cell lines proliferation inhibition was determined *via* Cell Titer Glo (Promega, Madison, WI, USA) according to the manufacturer's protocol.

### Immunoblot analysis

Total proteins from patient tumor derived cell lines were isolated using RIPA buffer (Sigma-Aldrich, St. Louis, MO, USA) containing a protease inhibitor cocktail (Roche, Mannheim, Germany) and phosphatase inhibitor cocktail (Roche), and protein concentrations were determined according to Bradford procedure using a Quick Start Bradford Protein Assay (Bio-Rad, Hercules, CA, USA). Thirty μg of proteins were subjected to 10% SDS-polyacrylamide gel electrophoresis, and electro-transferred onto nitrocellulose membranes. The membranes were blocked with 5% nonfat dry milk in Tris-buffered saline containing 0.1% v/v Tween 20, and probed overnight at 4°C with specific antibodies: pALK (Tyr 1586), ALK (C-19) from Santa Cruz biotechnology (Santa Cruz, CA, USA), and pERK1/2 (Thr202/Tyr204), ERK1/2 (Thr202/Tyr204) from Cell Signaling Technology (Beverly, MA, USA), and beta actin from Sigma Aldrich. Horseradish peroxidase-conjugated anti-rabbit or mouse IgG (Vector, Burlingame, CA, USA) were used as secondary antibodies, and signals were detected by chemiluminescence using ECL Western Blotting Substrate (Thermo Scientific, Rockford, IL, USA), and visualized by using LAS-4000 (Fujifilm, Tokyo, Japan).
